# Harnessing and Degradation Mechanism of Persistent Polyethylene Waste by Newly Isolated Bacteria from Waxworm and Termite Gut Symbionts

**DOI:** 10.3390/microorganisms13081929

**Published:** 2025-08-18

**Authors:** Sameh Samir Ali, Jianzhong Sun, Rania Al-Tohamy, Maha A. Khalil, Tamer Elsamahy, Michael Schagerl, Daochen Zhu, Shimaa El-Sapagh

**Affiliations:** 1 Biofuels Institute, School of the Environment and Safety Engineering, Jiangsu University, Zhenjiang 212013, China; samh_samir@science.tanta.edu.eg (S.S.A.); rony.altohamy@ujs.edu.cn (R.A.-T.); elsamahy@ujs.edu.cn (T.E.); zhu@ujs.edu.cn (D.Z.); 2 Botany and Microbiology Department, Faculty of Science, Tanta University, Tanta 31527, Egypt; shayma_elsabagh@science.tanta.edu.eg; 3 Department of Biology, College of Science, Taif University, P.O. Box 11099, Taif 21944, Saudi Arabia; maha.ak1@tu.edu.sa; 4 Department of Functional and Evolutionary Ecology, University of Vienna, Djerassiplatz 1, A-1030 Vienna, Austria

**Keywords:** persistent pollutants, plastic polymers, biodegradation, insect gut symbionts, bioremediation, ligninase-producing bacteria

## Abstract

Pollution from synthetic polymers, particularly low-density polyethylene (LDPE), poses a significant environmental challenge due to its chemical stability and resistance to degradation. This study investigates an eco-biotechnological approach involving bacterial strains isolated from insect guts—*Bacillus cereus* LDPE-DB2 (from *Achroia grisella*) and *Pseudomonas aeruginosa* LDPE-DB26 (from *Coptotermes formosanus*)—which demonstrate the ability to degrade LDPE, potentially through the action of lignin-modifying enzymes. These strains exhibited notable biofilm formation, enzymatic activity, and mechanical destabilization of LDPE. LDPE-DB2 exhibited higher LDPE degradation efficiency than LDPE-DB26, achieving a greater weight loss of 19.8% compared with 11.6% after 45 days. LDPE-DB2 also formed denser biofilms (maximum protein content: 68.3 ± 2.3 µg/cm^2^) compared with LDPE-DB26 (55.2 ± 3.1 µg/cm^2^), indicating stronger surface adhesion. Additionally, LDPE-DB2 reduced LDPE tensile strength (TS) by 58.3% (from 15.3 MPa to 6.4 ± 0.4 MPa), whereas LDPE-DB26 induced a 43.1% reduction (to 8.7 ± 0.23 MPa). Molecular weight analysis revealed that LDPE-DB2 caused a 14.8% decrease in weight-averaged molecular weight (Mw) and a 59.1% reduction in number-averaged molecular weight (Mn), compared with 5.8% and 32.7%, respectively, for LDPE-DB26. Fourier-transform infrared spectroscopy (FTIR), X-ray diffraction (XRD), and gel permeation chromatography (GPC) analyses revealed substantial polymer chain scission and crystallinity disruption. Gas chromatography–mass spectrometry (GC-MS) identified environmentally benign degradation products, including alkanes, alcohols, and carboxylic acids. This study demonstrates a sustainable route to polyethylene biotransformation using insect symbionts and provides insights for scalable, green plastic waste management strategies in line with circular economy goals.

## 1. Introduction

Plastic waste has emerged as one of the most critical environmental challenges of the modern era [1]. Since the advent of mass plastic production in the 1950s, approximately 8.3 billion tons of plastic resins, fibers, and products have been manufactured globally as of 2017 [2]. The global production of plastic has increased substantially each year, surpassing 380 million tons in 2015 alone [3]. However, the prevailing linear economic model of “take–make–dispose” has resulted in a disproportionately large fraction of this plastic becoming post-consumer waste [4]. Of all plastic waste generated since the 1950s, only about 14% has been recycled through conventional mechanical recycling processes [5]. Waste-to-energy technologies have incinerated approximately 12% of plastic waste, often releasing toxic byproducts into the atmosphere [6]. Meanwhile, an overwhelming 79% of plastic waste accumulates in municipal solid waste streams, leading to landfilling or unchecked environmental leakage [7].

The vast quantities of plastic waste that are landfilled or discarded into the environment pose severe ecological threats. An estimated 150 million tons of plastic debris currently circulate in the oceans [8], with every major ocean gyre and even remote shorelines worldwide now contaminated with microplastics—plastic fragments smaller than 5 mm [9]. On land, unregulated disposal has resulted in the contamination of extensive areas, significantly affecting wildlife in various ecosystems [7]. Additionally, leachates from aging plastic in landfills contribute to soil and water pollution through the release of toxic additives [10]. Without urgent intervention, plastic pollution in the oceans is projected to surpass the biomass of fish by 2050 [11]. Given that global plastic production continues to rise without adequate waste management infrastructure, the scale of the plastic crisis is set to escalate further in the coming decades. Therefore, innovative approaches are critically needed to establish a circular economy for plastic waste on a large scale.

Low-density polyethylene (LDPE) constitutes a significant fraction of discarded plastic waste streams [12]. As one of the most widely used plastics, LDPE is commonly found in packaging films, carrier bags, containers, bottles, and various consumer products due to its flexibility, affordability, and durability [13]. However, the same durability that makes LDPE valuable also presents substantial environmental risks post-consumer use. LDPE is highly resistant to degradation, with an estimated decomposition time exceeding a century under open environmental conditions [14]. Its non-biodegradability poses significant challenges for traditional waste management methods. Landfilling or indiscriminate dumping of LDPE waste results in long-term soil and water contamination, while incineration generates hazardous emissions. Moreover, mechanical recycling faces limitations due to contamination, downcycling to lower-quality materials, and high processing costs [15]. Given that plastic waste generation is projected to rise dramatically in the coming years, novel strategies for the efficient degradation of LDPE are urgently required [16].

One promising approach involves leveraging microbial biodegradation to break down polyethylene polymers. Various microorganisms, including bacteria, fungi, and actinomycetes, have demonstrated the ability to utilize polyethylene as a sole carbon source [17,18,19,20]. However, the biodegradation rates achieved thus far have been relatively low, limiting the practical applicability of microbial degradation. A potential solution lies in the isolation of more efficient polyethylene-degrading bacteria. In recent years, the gut microbiota of insects has been identified as a valuable source of plastic-degrading bacteria [21]. Insects that consume organic polymers, such as waxworms and termites, harbor specialized microbial communities in their digestive systems that produce enzymes capable of breaking down complex hydrocarbons. Among these, the lesser waxworm (*Achroia grisella*) and the wood-feeding termite (*Coptotermes formosanus*) have demonstrated remarkable potential for plastic biodegradation through their gut symbionts [12,17,22,23].

The gut symbionts of plastic-degrading insects play a crucial role in breaking down synthetic polymers through specialized enzymatic and metabolic pathways [24,25]. Insects such as waxworms, mealworms, termites, and beetle larvae harbor diverse microbial communities capable of degrading polyethylene, polystyrene, and other plastic materials [12,17,22,26,27]. These gut bacteria, including *Bacillus*, *Pseudomonas*, *Enterobacter*, and *Klebsiella* species, produce oxidative and hydrolytic enzymes such as laccases, peroxidases, lipases, and esterases, which initiate the breakdown of polymer chains. The degradation process often begins with microbial colonization of the plastic surface, followed by enzymatic oxidation that introduces functional groups, making the material more susceptible to further microbial metabolism [28]. Some gut bacteria also facilitate biofilm formation, enhancing adhesion to the plastic substrate and prolonging enzymatic activity [29]. In many cases, the degraded plastic fragments are further converted into simpler compounds, such as short-chain fatty acids and carbon dioxide, integrating the polymer residues into microbial metabolic pathways [30,31]. The discovery of these insect gut symbionts has opened new possibilities for bioremediation strategies, offering a sustainable and eco-friendly alternative to traditional plastic waste disposal methods. Understanding and optimizing these microbial interactions could lead to the development of enhanced bioaugmentation techniques and engineered microbial consortia for large-scale plastic degradation applications.

The lesser waxworm, *Achroia grisella*, is a species of pyralid moth larvae commonly found infesting beehives, where it feeds on beeswax and other organic materials [32]. The ability of *Achroia grisella* to digest wax is attributed to the enzymatic activity of its gut microbiota, which can also degrade synthetic polymers such as polyethylene [33]. Studies have shown that the gut bacteria of waxworms, including *Bacillus cereus* and other lignin-degrading microbes, play a crucial role in breaking down polyethylene through oxidative and hydrolytic mechanisms [12,34]. These bacteria secrete enzymes such as laccase (Lac), lignin peroxidase (LiP), and manganese peroxidase (MnP), which facilitate the breakdown of polyethylene into smaller, bioavailable fragments. The degradation process involves the oxidation of polyethylene chains, leading to the formation of carbonyl and hydroxyl groups, which are further metabolized by microbial activity [35,36]. This unique adaptation has positioned *Achroia grisella* as a promising model for biological plastic degradation, offering a sustainable solution to polymer waste management.

The Formosan subterranean termite, *Coptotermes formosanus*, is a highly destructive wood-feeding insect known for its ability to degrade lignocellulosic materials efficiently [37]. This capability is primarily attributed to its symbiotic relationship with a diverse microbial community residing in its gut. The termite gut harbors a consortium of bacteria and protists that specialize in breaking down complex polymers, including cellulose and lignin, through enzymatic hydrolysis and oxidation [38]. Notably, some of these gut symbionts, such as *Pseudomonas aeruginosa*, have demonstrated the ability to degrade synthetic plastics, including LDPE [18]. The degradation process involves the colonization of the plastic surface by microbial biofilms, followed by the secretion of oxidative enzymes that cleave polymer chains [39]. The metabolic byproducts are subsequently utilized by the microbial community as a carbon source, facilitating further breakdown [40]. Given the efficiency of *Coptotermes formosanus* symbionts in processing recalcitrant polymers, their application in plastic biodegradation presents a promising avenue for reducing plastic waste accumulation in the environment.

The discovery of plastic-degrading bacteria within the gut microbiota of *Achroia grisella* and *Coptotermes formosanus* highlights the potential of insect-derived microbial consortia in addressing plastic pollution. Harnessing these microbial capabilities through biotechnological approaches, such as enzyme isolation, genetic engineering, and bioaugmentation, could lead to the development of efficient and scalable plastic biodegradation systems. Future research should focus on optimizing the conditions for microbial degradation, characterizing the enzymatic pathways involved, and evaluating the environmental feasibility of large-scale applications. By leveraging the natural plastic-degrading abilities of insect gut symbionts [41], it may be possible to develop eco-friendly solutions for plastic waste management and contribute to global sustainability efforts.

Given the environmental persistence of LDPE and the limited efficiency of current degradation methods, we hypothesized that bacterial strains isolated from insect gut environments—specifically waxworms and wood-feeding termites—harbor metabolic capabilities adapted for hydrocarbon-rich diets that could be leveraged to enhance LDPE biodegradation. The persistence of LDPE in terrestrial and aquatic environments poses a major environmental concern due to its resistance to degradation and widespread use in consumer products. While several studies have reported microorganisms capable of degrading LDPE, the distribution of such microbial strains in diverse ecosystems remains limited, and the precise biochemical pathways involved in LDPE biodegradation are still not fully resolved. This gap underscores the need for continued investigation into novel microbial sources and detailed mechanistic analyses.

This study investigates the biodegradation potential of insect gut-derived bacterial strains (*Bacillus cereus* LDPE-DB2 and *Pseudomonas aeruginosa* LDPE-DB26) in depolymerizing LDPE, a widely used yet environmentally persistent synthetic polymer. The primary objective is to assess the strains’ ability to form biofilms, produce relevant extracellular enzymes, and induce structural degradation of LDPE, with the goal of establishing an ecotechnological foundation for microbial plastic waste degradation. By integrating physicochemical and analytical evaluations, this work aims to develop a scalable biocatalytic system aligned with sustainable environmental management practices. The findings offer valuable insights into microbial degradation pathways and could inform future strategies for bioaugmentation and the synthetic biology-based engineering of hyper-efficient plastic-degrading organisms, providing a promising approach to mitigating global plastic pollution.

## 2. Materials and Methods

### 2.1. LDPE Polymer, Bacterial Strains, and Culture Media

Flexible sheets of LDPE labware with no additives (25 µm thickness and 50% crystallinity) were purchased from ThermoFisher Scientific Co., Shanghai, China. The sheets were prepared as previously described [17]. LDPE sheets were washed with 70% ethanol for 30 min and allowed to dry for 12 h at 40 °C under sterile conditions in a laminar airflow cabinet. To check for potential contamination, randomly selected dried LDPE sheets were placed on nutrient agar plates and incubated at 30 °C for 48 h. No microbial growth was observed, confirming the absence of contamination prior to use in the biodegradation assays.

Two previously isolated bacterial strains, *Bacillus cereus* LDPE-DB2 (from the gut symbionts of the plastic-eating lesser waxworm, *Achroia grisella*) and *Pseudomonas aeruginosa* LDPE-DB26 (from the gut symbionts of the wood-feeding termite, *Coptotermes formosanus*) [12,17], were employed for LDPE biodegradation by incubating the flasks at 30 °C for up to 30 days with an agitation speed of 120 rpm. These bacterial strains were identified based on 16S rRNA gene sequencing [12]. The identification was performed using standard molecular techniques, and sequence alignment was carried out using BLAST (Basic Local Alignment Search Tool, version 2.14.0+) against the NCBI nucleotide database. *Bacillus cereus* LDPE-DB2 and *Pseudomonas aeruginosa* LDPE-DB26 strains were selected for their ability to produce ligninolytic enzymes, including Lac, MnP, and LiP.

The liquid carbon-free basal medium (LCFBM) was prepared following the American Society for Testing and Materials standard [42] to determine the efficiency of bacterial strains in degrading LDPE polymer as a sole carbon source. LDPE sheets (1 cm × 1 cm; approximately 40 mg per sheet) were used as the polymer substrate. For each experimental replicate, one LDPE sheet was added to 100 mL of sterilized LCFBM medium. The LCFBM consists of MgSO_4_·7H_2_O (1.0 g/L), NH_4_NO_3_ (1.0 g/L), KH_2_PO_4_ (0.7 g/L), MnSO_4_·H_2_O (0.001 g/L), K_2_HPO_4_ (0.7 g/L), ZnSO_4_·H_2_O (0.001 g/L), NaCl (0.002 g/L), and FeSO_4_·7H_2_O (0.002 g/L). For the preparation of the carbon-free basal agar medium (CFBAM), 15 g/L of agar was added to the content of LCFBM mentioned above. As a control, LDPE sheets were incubated in sterile LCFBM medium without the addition of bacterial strains under identical conditions. These controls were processed in parallel and subjected to the same washing, incubation, and analytical procedures as the experimental treatments to assess abiotic changes in LDPE and ensure that observed effects were due to microbial activity. 

### 2.2. Assessment of Cell Surface Hydrophobicity

The bacterial adhesion to hydrocarbons (BATH) assay was employed to evaluate the hydrophobic character of LDPE-DB2 and LDPE-DB26, following the protocol defined by Harshvardhan and Jha [43]. Active log-phase cultures of each strain were initially centrifuged at 5000 rpm to pelletize cells. The resulting pellets were twice resuspended in phosphate urea magnesium (PUM) buffer consisting of 7.26 g/L KH_2_PO_4_, 1.8 g/L urea, 17 g/L K_2_HPO_4,_ and 0.2 g/L MgSO_4_, to thoroughly wash bacterial cells to reach a cell density (OD_600_) of 1.2. Aliquots of 1.2 mL from each standardized culture were individually combined with increasing volumes of hexadecane (ranging from 0 to 300 μL) within separate test tubes. Samples were vortex-mixed for 10 min to enable cell-hydrocarbon interactions, followed by a 2 min separation phase. The OD_600_ of resultant aqueous layers was subsequently measured spectrophotometrically against a PUM buffer blank as a control.

### 2.3. LDPE Biodegradation Assay

Viability of bacteria and biofilm biomass evaluation on LDPE surface was conducted. This involved carefully removing the LDPE sheet from the liquid medium and rinsing it with distilled water to release loosely attached bacteria. Subsequently, the LDPE films were sonicated in a water bath with 1 mL of 0.9% saline solution to dislodge the bacterial biofilm. A portion of this solution was then serially diluted and plated onto NA, which was subsequently incubated at 35 °C for 48 h. The results were recorded as CFU, indicating the number of viable bacterial cells. The total protein content of bacterial biofilms attached to LDPE sheets was determined using the Bradford assay [18]. After sonication and collection of biofilm suspensions, 100 µL of each sample was mixed with 1 mL of Bradford reagent (Bio-Rad, Hercules, CA, USA), incubated at room temperature for 10 min, and the absorbance was measured at 595 nm using a spectrophotometer. Bovine serum albumin (BSA) was used to generate a standard calibration curve. All measurements were performed in triplicate. At each time point (every 5 days), a separate set of LDPE films was removed from the culture medium for analysis. The films were gently washed with distilled water to eliminate medium residues or loosely attached cells. For each time point, three replicate LDPE films were analyzed (*n* = 3). The bacterial biomass obtained from the LDPE sheet was subjected to water bath sonication for 3 min in 1 mL of 0.9% saline. The weight loss percentage of PE films was determined using the following equation:

(1) $$Biodegradation\;(\%)=\frac{\left(Initial\;weight-final\;weight\right)\times 100}{initial\;weight}$$

The rate of LDPE degradation by the microbial isolates was modeled using a first-order kinetic model:

(2) $$-Kt=\ln\frac{Ⱳt}{Ⱳ0}$$

where K represents the rate constant for polymer depletion per day, t refers to the time in days, Wt is the weight of remaining LDPE in grams at time t, and W0 denotes the initial weight of LDPE in grams. After calculating the K value through kinetic modeling, the half-life (t1/2) of LDPE degradation was then determined using the following equation [44]:

(3) $$t\frac{1}{2}=\frac{ln\left(2\right)}{K}$$

The tensile strength (TS) of LDPE films was measured using a universal testing machine (e.g., Instron 3365, Grove City, PA, USA) equipped with a 500 N load cell. Rectangular strips of LDPE (50 mm × 10 mm) were cut from treated and control films. Measurements were performed at room temperature with a crosshead speed of 50 mm/min and an initial grip separation of 30 mm. All measurements were conducted in triplicate at regular 5-day intervals and compared with the initial TS value of the untreated plastic sheet and average values were reported.

### 2.4. Characterization of LDPE Films

In order to eliminate microbial biomass from the LDPE surface, the treated films were dried and subsequently rinsed with sodium dodecyl sulfate (2%) and distilled water for 15 min. LDPE sheets were fixed for 2 h in glutaraldehyde (4%) at 4 °C and dehydrated for 30 min with ethanol (50%). The LDPE sheets were incubated overnight in ethanol (70%) at 25 °C, coated with gold for 40 s, and scanned through a scanning electron microscope (SEM, Hillsboro, OR, USA) to explore the changes in LDPE surface topography before and after biological treatment. X-ray diffraction analysis (XRD, Siemens Analytical X-Ray Instruments, Inc., Madison, WI, USA) is automatically operated (λ = 1.5418 A) by Cu Kα radiation. The scattered radiation was operated in the angular interval (2Ɵ) from 10 to 90°.

Changes in the functional groups and surface structure of the LDPE sheet were analyzed by Fourier Transform Infrared (FTIR) (PerkinElmer, Shelton, CT, USA) in the frequency range of 400–4000 cm^−1^. The crystallinity percentage of the LDPE sheet was detected using XRD analysis following the method described by Torre et al. [45] and calculated using the following formula:

(4) $$Crystallinity\left(\%\right)=100-\left[\frac{1-Aa/1.233Ab}{1+(Aa/Ab)}\times 100\right]$$

where Aa is the absorbance at 1473 and Ab is the absorbance at 1463.

Molecular weight analysis of LDPE residues was performed using high-temperature gel permeation chromatography (HT-GPC; PL-GPC 220, Agilent Technologies, Santa Clara, CA, USA). Approximately 5 mg of treated and control LDPE films was dissolved in 5 mL of 1,2,4-trichlorobenzene (TCB) containing 0.0125% (*w*/*v*) butylated hydroxytoluene (BHT) as antioxidant. Samples were stirred at 160 °C for 2 h to ensure complete dissolution. The separation was carried out at 160 °C using a PLgel Olexis mixed-bed column (300 mm × 7.5 mm) with a flow rate of 2.0 mL/min. The instrument was calibrated using narrow polystyrene standards, and data processing was performed with Agilent GPC/SEC software version 5.03. For GC-MS analysis of the metabolites, samples were extracted from the culture supernatant using dichloromethane (DCM) at a 1:1 volume ratio, followed by concentration under nitrogen. One microliter of each extract was injected into an Agilent 7890B gas chromatograph coupled with an Agilent 5977B mass selective detector (MSD). A DBS-MS column (30 m × 0.25 mm i.d., 0.25 μm film thickness; J&W Scientific, Folsom, CA, USA) was used for separation. The oven program was set as follows: initial temperature 50 °C (held for 2 min), increased at 10 °C/min to 300 °C, and held for 10 min. The injector and detector temperatures were set to 280 °C. Helium was used as the carrier gas at a constant flow rate of 1.0 mL/min. The mass spectrometer operated in electron ionization (EI) mode at 70 eV, scanning from *m*/*z* 40 to 550.

### 2.5. Statistical Analysis

Data are presented as mean ± standard deviation (SD). The data were analyzed using Origin 2019 and GraphPad Prism version 8.0.2. The variation between groups was assessed by one-way ANOVA and Tukey test at a probability of *p*-value less than 0.05, and significance levels are indicated as follows: * *p* ≤ 0.05, ** *p* ≤ 0.01, *** *p* ≤ 0.001, and **** *p* ≤ 0.0001, while ns indicates no statistically significant difference (*p* > 0.05).

## 3. Results and Discussion

The discovery of plastic-degrading bacteria in insect guts holds immense potential for biodegradation applications. Industrial-scale solutions, such as biofilm-based degradation systems, can be developed to harness these microbes for waste management. Furthermore, it is essential to assess the long-term ecological effects of insect-mediated plastic biodegradation to ensure sustainable implementation.

### 3.1. Bacterial Cell Hydrophobicity

**Figure 1:**
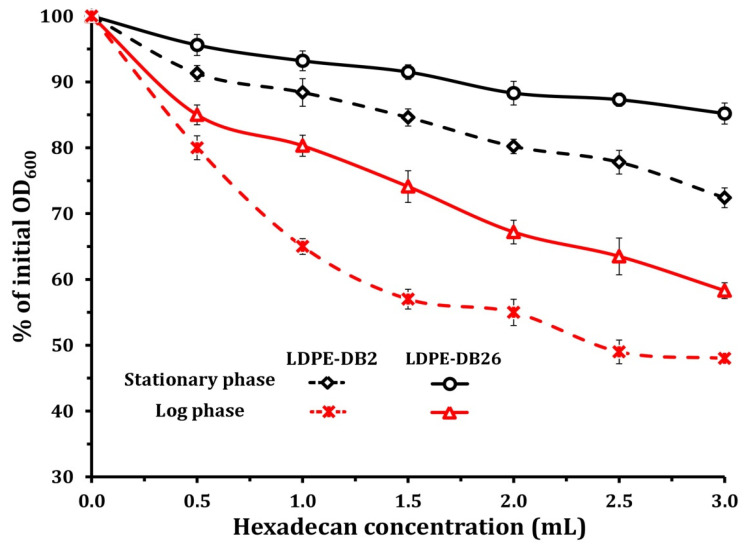
*B. cereus* LDPE-DB2 and *P. aeruginosa* LDPE-DB26 hydrophobicity assessment using the bacterial adhesion to hydrocarbon assay.

Figure 1 illustrates the hydrophobicity assessment of *Bacillus cereus* LDPE-DB2 and *Pseudomonas aeruginosa* LDPE-DB26 using the BATH assay. The graph shows the percentage of initial OD_600_ as a function of hexadecane concentration (mL) for bacterial cells in both the stationary phase and log phase. The extent of bacterial adhesion to hexadecane reflects their cell surface hydrophobicity, with a greater reduction in OD_600_ indicating stronger hydrophobic interactions. The results reveal a clear difference in hydrophobicity between the two bacterial strains and across different growth phases. *Bacillus cereus* LDPE-DB2 exhibits a significantly greater reduction in OD_600_ than *Pseudomonas aeruginosa* LDPE-DB26 across all hexadecane concentrations, suggesting that *Bacillus cereus* LDPE-DB2 has a more hydrophobic cell surface. Furthermore, both strains show higher hydrophobicity in the log phase compared with the stationary phase, as indicated by the steeper decline in OD_600_ in log-phase cells. This suggests that bacterial hydrophobicity is growth-phase dependent, with cells in the log phase displaying enhanced surface hydrophobicity due to increased expression of outer membrane proteins and biosurfactants that facilitate adhesion to hydrophobic substrates such as LDPE.

As the hexadecane concentration increases, the OD_600_ reduction becomes more pronounced, indicating stronger bacterial attachment to the hydrocarbon phase. At 3.0 mL of hexadecane, *Bacillus cereus* LDPE-DB2 in the log phase exhibits the highest hydrophobicity, with OD_600_ dropping below 40% of its initial value, compared with ~55% for *Pseudomonas aeruginosa* LDPE-DB26 in the log phase. In the stationary phase, both strains exhibit less pronounced hydrophobicity, with OD_600_ remaining above 70% for *Pseudomonas aeruginosa* LDPE-DB26 and 60% for *Bacillus cereus* LDPE-DB2. These results suggest that *Bacillus cereus* LDPE-DB2 adheres more efficiently to hydrophobic surfaces like LDPE, which may contribute to its superior biofilm formation and LDPE degradation efficiency compared with *Pseudomonas aeruginosa* LDPE-DB26. The observed differences in hydrophobicity between the two strains can be attributed to variations in cell surface properties, extracellular polymeric substances production, and membrane composition. *Bacillus cereus* LDPE-DB2’s stronger hydrophobic interactions likely enhance its colonization of LDPE surfaces, facilitating biofilm formation and polymer degradation. Conversely, *Pseudomonas aeruginosa* LDPE-DB26’s relatively lower hydrophobicity suggests a weaker initial attachment to the polymer surface, which may explain its comparatively lower LDPE degradation efficiency. Overall, the findings from Figure 1 suggest that *Bacillus cereus* LDPE-DB2 possesses a greater capacity for LDPE surface colonization, owing to its higher cell surface hydrophobicity, particularly in the log phase. This characteristic likely contributes to its superior biodegradation performance compared with *Pseudomonas aeruginosa* LDPE-DB26.

The insect gut microbiome has emerged as a promising reservoir for bacteria capable of degrading persistent plastic polymers such as LDPE. Insects such as the lesser waxworm (*Achroia grisella*) and wood-feeding termites harbor symbiotic microbial communities within their digestive tracts that play essential roles in breaking down recalcitrant natural polymers like lignocellulose and wax [22,25]. These microbial consortia have adapted to thrive in nutrient-poor and structurally complex environments, equipping them with enzymatic machinery and physicochemical traits—such as enhanced hydrophobicity—that are favorable for plastic colonization and degradation. The ability of gut-associated bacteria to adhere to hydrophobic substrates like LDPE is particularly significant, as adhesion is a prerequisite for effective biofilm formation and subsequent polymer breakdown [46]. Gut-derived strains, such as *Bacillus cereus* LDPE-DB2 isolated in this study, often exhibit high surface hydrophobicity, likely reflecting their ecological niche within the gut, where interaction with waxy and lipid-like substances is common. This trait enhances their capacity to colonize and form stable biofilms on synthetic hydrophobic materials. Therefore, the strong hydrophobicity and superior adhesion capabilities observed in *Bacillus cereus* LDPE-DB2 can be attributed not only to its surface properties but also to its ecological origin from the insect gut environment. The ability of microorganisms to utilize a substrate as a carbon and energy source depends on their capacity to adhere to that substrate. Previous studies have reported that the interaction between bacterial cells and hydrocarbons, such as LDPE, is influenced by several physical parameters. These include the forces facilitating bacterial attachment to solid surfaces, substrate characteristics, and microbial properties [47]. Generally, hydrophobic microorganisms preferentially colonize hydrophobic surfaces, while hydrophilic bacteria favor hydrophilic materials. Colonization is a critical step for microbial utilization of any substrate. The adhesion of bacteria to hydrophilic or hydrophobic substrates is influenced by multiple factors, including the attachment mechanisms of the microbe and the properties of both the substrate and the organism. Due to the inherently hydrophobic nature of LDPE, microorganisms with more hydrophobic cell surfaces are likely to exhibit stronger interactions with this plastic polymer [48]. These findings reinforce the potential of insect gut bacteria as valuable agents in the biodegradation of plastics and support the observed correlation between bacterial hydrophobicity and LDPE degradation efficiency.

### 3.2. Bacterial Growth and Biofilm Biomass Evaluation on LDPE Surface

**Figure 2:**
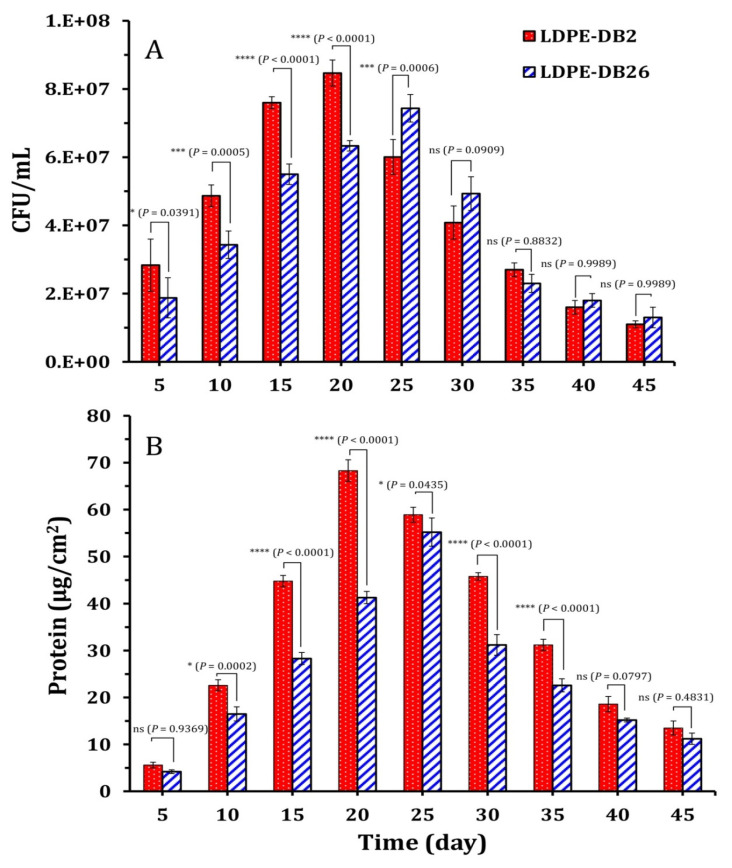
Bacterial responses to LDPE-containing medium: (**A**) Growth of bacterial planktonic cells in LDPE-containing medium. (**B**) Biofilm protein content of bacteria cultured in LDPE-containing LCFBM. Significance levels are indicated as follows: * *p* ≤ 0.05, *** *p* ≤ 0.001, and **** *p* ≤ 0.0001, while ns indicates no statistically significant difference (*p* > 0.05).

Figure 2 illustrates the growth of *Bacillus cereus* LDPE-DB2 and *Pseudomonas aeruginosa* LDPE-DB26 on LDPE-containing medium (Figure 2A) and their respective biofilm protein content (Figure 2B). The bacterial growth, measured in CFU/mL, and biofilm biomass, assessed via total protein content, were tracked over 45 days. *Bacillus cereus* LDPE-DB2 reached its maximum growth (8.5 × 10^7^ CFU/mL) by the 20th day, while *Pseudomonas aeruginosa* LDPE-DB26 achieved its maximum growth (7.4 × 10^7^ CFU/mL) by the 25th day. The results were statistically analyzed using two-way ANOVA for multiple comparisons between the two strains. The analysis revealed that bacterial growth after the 30th day did not change significantly (*p* < 0.05), as illustrated in Figure 2A. However, the growth of LDPE-DB2 from the 5th to the 30th day was significantly higher than that of LDPE-DB26 (*p* < 0.05).

In this study, biofilm formation and growth of LDPE-DB2 and LDPE-DB26 on the LDPE sheet were evaluated by quantifying the total protein content of the bacterial biomass colonizing the LDPE surface. As depicted in Figure 2B, the protein content increased gradually during biological treatment, peaking on the 20th and 25th days for LDPE-DB2 (68.3 ± 2.3 μg/cm^2^) and LDPE-DB26 (55.2 ± 3.1 μg/cm^2^), respectively. This represented a significant (*p* < 0.0001) increase compared with the protein content measured on the 5th day (5.6 ± 0.6 μg/cm^2^ for LDPE-DB2 and 4.2 ± 0.4 μg/cm^2^ for LDPE-DB26). However, by the end of the incubation period, the microbial biomass decreased significantly (*p* < 0.05), reaching 13.5 ± 1.4 μg/cm^2^ for LDPE-DB2 and 11.2 ± 1.3 μg/cm^2^ for LDPE-DB26. This decline may be attributed to the detachment of microbial cells from the LDPE surface due to prolonged incubation.

The ability of bacteria to colonize and form biofilms on hydrophobic surfaces such as LDPE is essential for initiating and sustaining plastic biodegradation, especially under nutrient-limited conditions. In this context, insect gut microbiota—particularly those of larvae like the lesser waxworm and wood-feeding termites—are increasingly recognized as rich sources of plastic-degrading bacteria with robust biofilm-forming capabilities. These insects naturally ingest complex and recalcitrant substrates such as lignin, cellulose, and beeswax, which share structural and chemical similarities with synthetic polymers like polyethylene. As a result, their gut-associated bacteria are adapted to thrive in harsh environments, often under carbon-limiting conditions, and exhibit enzymatic activities and surface properties that facilitate adhesion to and breakdown of hydrophobic materials. For instance, *Bacillus cereus* LDPE-DB2, isolated from such an insect gut environment, demonstrated significantly higher growth and biofilm biomass on LDPE surfaces compared with *Pseudomonas aeruginosa* LDPE-DB26. This superior performance can be attributed to the evolutionary adaptation of insect gut microbes to metabolize structurally resistant compounds and form stable biofilms in carbon-starved niches [49]. The prolonged biofilm viability observed in this study is consistent with previous findings on gut-derived microbes that maintain metabolic activity over extended periods, even in the absence of external carbon sources [50].

The results demonstrate the ability of both LDPE-DB2 and LDPE-DB26 to form biofilms and grow on the LDPE surface. However, LDPE-DB2 exhibited superior growth and biofilm formation compared with LDPE-DB26. Biofilm formation on plastic surfaces under carbon starvation is a complex process that varies among bacterial species. In this study, polyethylene films served as both a substrate for attachment and biofilm formation by *Bacillus cereus* and *Pseudomonas aeruginosa*, as well as the sole carbon source in a carbon-starved medium [12,51,52]. This led to the development of a thick biofilm strongly adhering to the LDPE surface, likely due to partial polymer degradation over time. Similarly, the ability of LDPE-DB2 and LDPE-DB26 to maintain an active biofilm on LDPE for 45 days may be linked to their consumption of low-molecular-weight substances within the polymer. These findings suggest that carbon starvation may enhance biofilm development on hydrophobic polymers like polyethylene. A similar biofilm formation pattern was observed with *Pseudomonas aeruginosa* ISJ14, which can colonize and form biofilms on LDPE films under varying media and growth conditions. This strain can also alter surface hydrophobicity in response to carbon-starved conditions [18]. Gilan and Sivan [53] reported that *Rhodococcus ruber* biofilms on polyethylene retained high viability even after 45 days without external carbon supplementation. These observations highlight the need for further exploration of the relationship between carbon availability, surface chemistry, and bacterial biofilm formation. Moreover, these insights underscore the ecological advantage of insect gut-associated bacteria and support their potential application in plastic waste bioremediation, particularly through enhanced biofilm formation on polymeric substrates like LDPE.

### 3.3. Biodegradation Assays

#### 3.3.1. Weight Loss of LDPE Films

**Figure 3:**
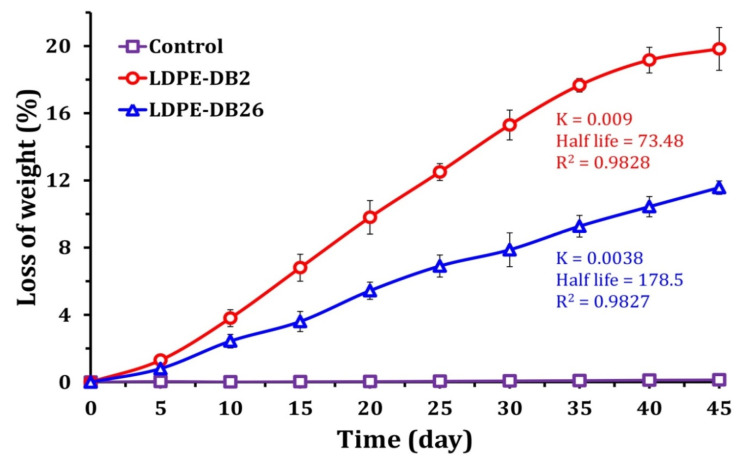
Changes in LDPE weight during the biodegradation process for 45 days.

Figure 3 depicts the percentage weight loss of LDPE films incubated with *Bacillus cereus* LDPE-DB2, *Pseudomonas aeruginosa* LDPE-DB26, and a control. The control film shows negligible weight loss, confirming that abiotic factors alone did not contribute significantly to polymer degradation. In contrast, the bacterial-treated LDPE films exhibit a progressive increase in weight loss over time, with *Bacillus cereus* LDPE-DB2 demonstrating a significantly higher degradation rate than *Pseudomonas aeruginosa* LDPE-DB26. After 45 days, *Bacillus cereus* LDPE-DB2 achieved a weight loss of 19.8%, whereas *Pseudomonas aeruginosa* LDPE-DB26 resulted in only 11.6% degradation, indicating that *Bacillus cereus* LDPE-DB2 is more effective in degrading LDPE. The degradation kinetics follow a first-order reaction model, with rate constants (K) and half-life values provided for each strain. The rate constant for LDPE-DB2 is 0.009 per day, resulting in a half-life of 73.48 days, whereas LDPE-DB26 has a lower rate constant of 0.0038 per day and a longer half-life of 178.5 days. These values confirm that *Bacillus cereus* LDPE-DB2 degrades LDPE at a significantly faster rate than *Pseudomonas aeruginosa* LDPE-DB26, likely due to its superior biofilm formation and enzymatic activity. LDPE-DB2 exhibited a 70.7% greater reduction in LDPE weight compared with LDPE-DB26 at the end of the incubation period. This enhanced degradation by LDPE-DB2 may be attributed to a combination of factors, including its higher cell surface hydrophobicity and greater biofilm formation ability, although differences in enzyme activity between the strains may also play a role.

Similarly, *Pseudomonas aeruginosa* ISJ14 demonstrated effective LDPE film degradation, with degradation efficiencies ranging from 6.5% to 8.7%, half-lives of 363 to 577.5 days, and removal constants of 0.0012 to 0.0019 per day, depending on the culture medium [18]. Other studies have reported that *Rhodococcus* sp. 36 and *Bacillus* sp. 27 degraded polystyrene with removal constants of 0.002 and 0.001 per day and half-lives of 346.5 and 693 days, respectively [44]. The kinetic analysis of LDPE-DB2 and LDPE-DB26 growth demonstrated their colonization on the LDPE surface and subsequent weight reduction, attributed to the consumption of LDPE as the sole carbon source. Comparable results were reported by Kyaw et al. [54], who observed a 20% reduction in LDPE film treated with *Pseudomonas aeruginosa* after 120 days. Soleimani et al. [55] found that *Streptomyces alborgiseolus* IR-SGS-T10 and *Streptomyces* sp. IR-SGS-T5 degraded 9.5 and 5.3% of LDPE film after 60 days, respectively. In contrast, a lower degradation rate was reported by Harshvardhan and Jha [43], who observed that *Kocuria palustris* M16, *Bacillus pumilus* M27, and *Bacillus subtilis* H1584, isolated from pelagic waters, degraded LDPE by 1.0%, 1.5%, and 1.75%, respectively, after 45 days of incubation.

Several insect species have demonstrated plastic-degrading capabilities through their gut symbionts, providing valuable insights into how microbial communities contribute to polymer breakdown. Mealworms (*Tenebrio molitor*) have been shown to consume and degrade polystyrene, with their gut bacteria metabolizing polystyrene into carbon dioxide and biomass. Research by Matyakubov and Lee [56] found that mealworms could degrade 56% of ingested polystyrene, a process facilitated by *Exiguobacterium* and *Pseudomonas* species. While *Tenebrio molitor* gut bacteria primarily target polystyrene, some isolates have also exhibited activity against polyethylene, though to a lesser extent than observed in this study’s LDPE degradation. Similarly, superworms (*Zophobas atratus*) have been reported to degrade polystyrene, with studies suggesting that *Klebsiella* and *Pseudomonas* species in their gut microbiome play a significant role in polymer degradation [57,58]. This finding aligns with our results, as *Pseudomonas aeruginosa* LDPE-DB26 exhibited moderate LDPE degradation, consistent with reports of *Pseudomonas* spp. in superworm studies. Beetle larvae (*Plodia interpunctella*), commonly known as the Indian meal moth, have demonstrated the ability to consume and digest polyethylene. The gut microbiome of *Plodia interpunctella* contains *Enterobacter* and *Bacillus* species, which secrete plastic-degrading enzymes [59,60]. Compared with the degradation efficiency observed in *Achroia grisella*, *Plodia interpunctella* larvae exhibit a slightly lower breakdown rate, likely due to differences in gut microbiota composition. Darkling beetles (*Alphitobius diaperinus*) have also shown potential in degrading polystyrene and polyethylene, with *Bacillus* and *Enterobacter* species identified as key contributors to plastic breakdown [24]. This supports our findings that *Bacillus cereus* plays a crucial role in polyethylene degradation, as observed in the gut microbiota of waxworms. These findings collectively indicate that bacterial isolates can degrade LDPE, and the degradation efficiency can be enhanced through the use of robust strains and optimized cultivation conditions.

The consistent findings across various insect species highlight the crucial role of insect gut microbiota in plastic degradation. Isolating and characterizing these microbial communities and their enzymes can pave the way for innovative biotechnological applications in plastic waste management. Future research should prioritize identifying and characterizing the specific enzymes involved in plastic degradation to enhance their activity and stability for industrial applications. Additionally, developing microbial consortia that replicate the synergistic interactions found in insect guts could improve degradation efficiency. Genetic engineering may also be utilized to enhance the plastic-degrading capabilities of these microbes or to transfer these abilities to other microorganisms suitable for large-scale applications. By harnessing the natural plastic-degrading potential of insect gut symbionts, we can develop sustainable and efficient strategies to combat plastic pollution.

#### 3.3.2. Tensile Strength Reduction in LDPE Film

**Figure 4:**
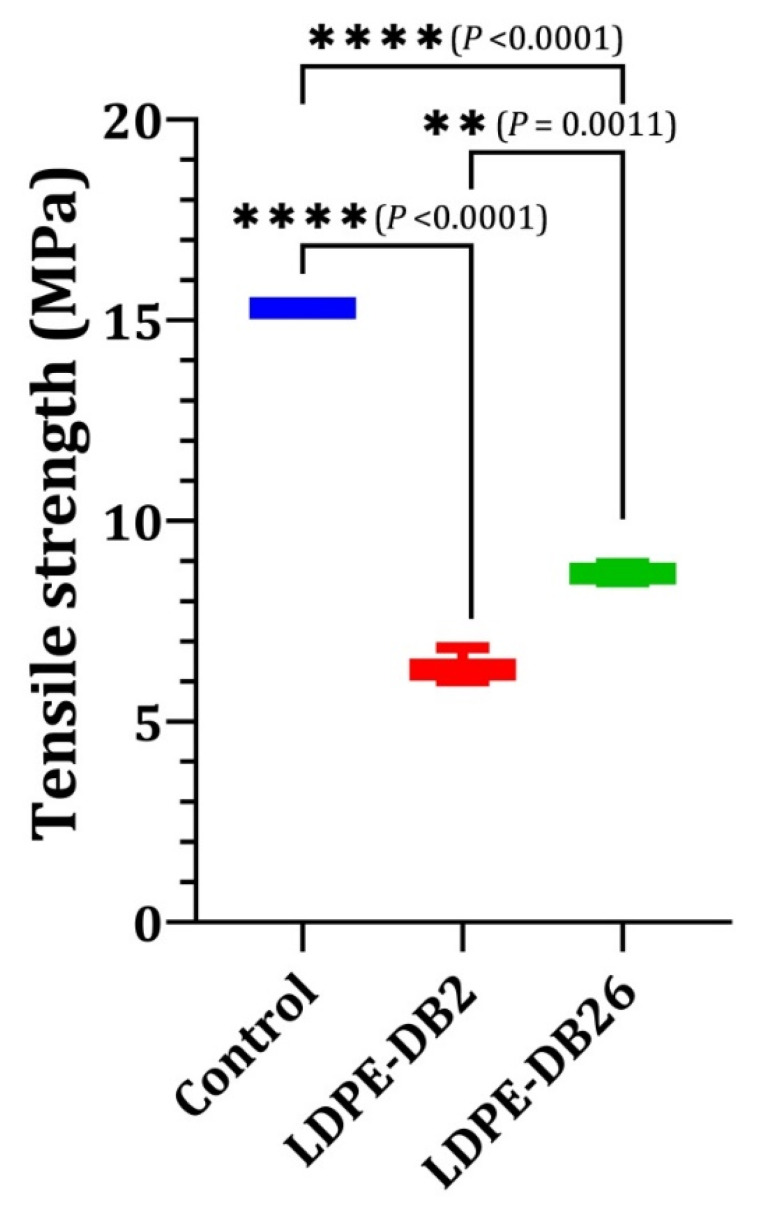
Changes in LDPE tensile strength during the biodegradation process for 45 days. Significance levels are indicated as follows: ** *p* ≤ 0.01, and **** *p* ≤ 0.0001.

Figure 4 shows the changes in tensile strength (TS) of LDPE films after microbial treatment in order to evaluate the ability of *Bacillus cereus* LDPE-DB2 and *Pseudomonas aeruginosa* LDPE-DB26 to degrade LDPE. The control LDPE film retains its original TS (~15.3 MPa), demonstrating minimal structural changes. However, both bacterial strains significantly reduce the TS of LDPE, confirming their ability to alter the polymer’s mechanical properties through enzymatic degradation. The TS of LDPE-DB2-treated films decreases to 6.4 ± 0.4 MPa (a 58.3% reduction), while LDPE-DB26-treated films retain a higher strength of 8.7 ± 0.23 MPa (a 43.1% reduction). Statistical analysis (ANOVA) confirms that these reductions are highly significant (*p* < 0.0001 for both bacterial strains compared with the control), with LDPE-DB2 causing a significantly greater reduction than LDPE-DB26 (*p* = 0.0011). The greater reduction in TS in LDPE-DB2-treated films aligns with its higher weight loss and more aggressive biodegradation activity, further reinforcing its superior performance in LDPE degradation. The reduction in TS of LDPE film by various bacterial strains has been documented in previous studies. For instance, *Citrobacter freundii* caused a 51.3% reduction in TS [12]. Similarly, Soleimani et al. [55] reported that *Streptomyces alborgiseolus* IR-SGS-T10 and *Streptomyces* sp. IR-SGS-T5 reduced the TS of LDPE by 46.3% and 46.7%, respectively. These findings demonstrate that these *Streptomyces* strains significantly impact the mechanical properties of LDPE, further supporting the ability of bacterial strains to alter the structural integrity of LDPE films. *Bacillus cereus* LDPE-DB2 exhibits significantly greater LDPE degradation efficiency than *Pseudomonas aeruginosa* LDPE-DB26, as evidenced by its higher weight loss, faster degradation kinetics, and greater reduction in TS. These differences are likely due to variations in biofilm formation, enzyme production, and metabolic pathways, which favor *Bacillus cereus* LDPE-DB2 as a more effective candidate for LDPE degradation applications.

The substantial reduction in TS observed in LDPE films treated with *Bacillus cereus* LDPE-DB2 further supports the hypothesis that gut-associated bacteria from insects are uniquely equipped for efficient plastic degradation. Insects such as the lesser waxworm and wood-feeding termites possess gut environments that have co-evolved with microbial communities specialized in breaking down resilient natural polymers like lignin, cellulose, and wax esters—materials that share physicochemical traits with synthetic polymers like LDPE. These gut symbionts are not only adapted to survive under nutrient-limited and anaerobic conditions but also possess diverse enzymatic systems capable of oxidizing and hydrolyzing high-molecular-weight compounds [25]. *Bacillus cereus* LDPE-DB2, likely originating from such an ecological niche, demonstrated a significantly greater reduction in LDPE TS compared with *Pseudomonas aeruginosa* LDPE-DB26. This superior mechanical degradation performance is likely a result of more robust enzyme secretion, deeper penetration into the polymer matrix, and enhanced metabolic efficiency—traits frequently associated with gut-derived microbes. Moreover, the enzymatic arsenal present in insect gut bacteria, including esterases, oxidoreductases, and hydrolases, can catalyze the scission of carbon–carbon and carbon–hydrogen bonds in polyethylene chains, thereby reducing the structural integrity of the polymer [61]. These findings underscore the biotechnological promise of insect gut bacteria in bioplastic degradation and offer mechanistic insight into how their unique metabolic capabilities translate into measurable alterations in polymer properties, such as TS. The integration of such microbes into bioremediation strategies may significantly enhance the breakdown of persistent plastic pollutants like LDPE.

### 3.4. Characterization of LDPE Film After Biodegradation

To elucidate the biodegradation pathway of LDPE sheets by *Bacillus cereus* LDPE-DB2 (isolated from waxworms) and *Pseudomonas aeruginosa* LDPE-DB26 (isolated from termite guts), analyses including HT-GPC, XRD, and FTIR were performed.

#### 3.4.1. Molecular Weight Reduction in LDPE Sheet

**Figure 5:**
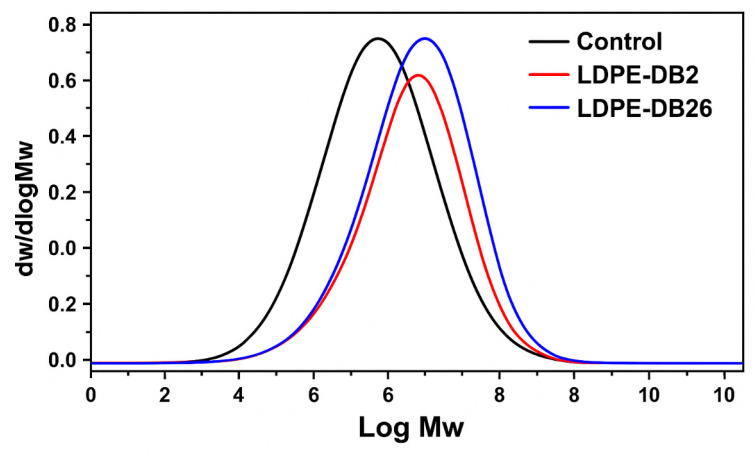
The molecular weight distribution curve of the LDPE treated with LDPE-DB2 and LDPE-DB26 versus the control after 45 days of incubation.

HT-GPC analysis was performed to assess the relative molecular weight and distribution of LDPE film samples. The results reveal clear shifts in molecular weight distribution between the treated and untreated LDPE films (Figure 5). The original (control) LDPE film exhibited a weight-averaged molecular weight (Mw) of 234.0 kDa and a number-averaged molecular weight (Mn) of 57.3 kDa. After 45 days of microbial treatment, the LDPE film incubated with LDPE-DB2 showed a substantial reduction in Mw to 199.3 kDa (14.8% decrease) and Mn to 23.3 kDa (59.1% decrease). In comparison, LDPE-DB26 treatment led to a reduction in Mw to 220.4 kDa (5.8%) and Mn to 38.3 kDa (32.7%). The polydispersity index (PDI) increased to 8.6 and 5.8 for LDPE-DB2 and LDPE-DB26, respectively, compared with 4.1 for the control, indicating enhanced heterogeneity in the treated films. The control LDPE exhibits a peak at a higher log Mw value, indicating the presence of high-molecular-weight polymer chains. However, after bacterial treatment, the peaks for both LDPE-DB2 and LDPE-DB26 shift toward lower molecular weights, confirming polymer fragmentation due to microbial degradation. The shift is more pronounced for LDPE-DB2-treated films, indicating that *Bacillus cereus* LDPE-DB2 induced greater polymer cleavage compared with *Pseudomonas aeruginosa* LDPE-DB26. The observed reduction in Mw suggests that bacterial enzymatic activity led to the breakdown of polyethylene chains into smaller fragments, supporting previous findings that LDPE-DB2 has a higher biodegradation efficiency than LDPE-DB26. These results demonstrate that LDPE-DB2, isolated from waxworms, exhibited more efficient modification of LDPE films than LDPE-DB26, isolated from termite guts. Similar findings were reported by Mohammadi et al. [62], who observed reductions in Mw and Mn values after biodegradation of LDPE using a bacterial consortium of *Bacillus* sp. and *Enterobacter asburiae*.

#### 3.4.2. Surface Morphology

**Figure 6:**
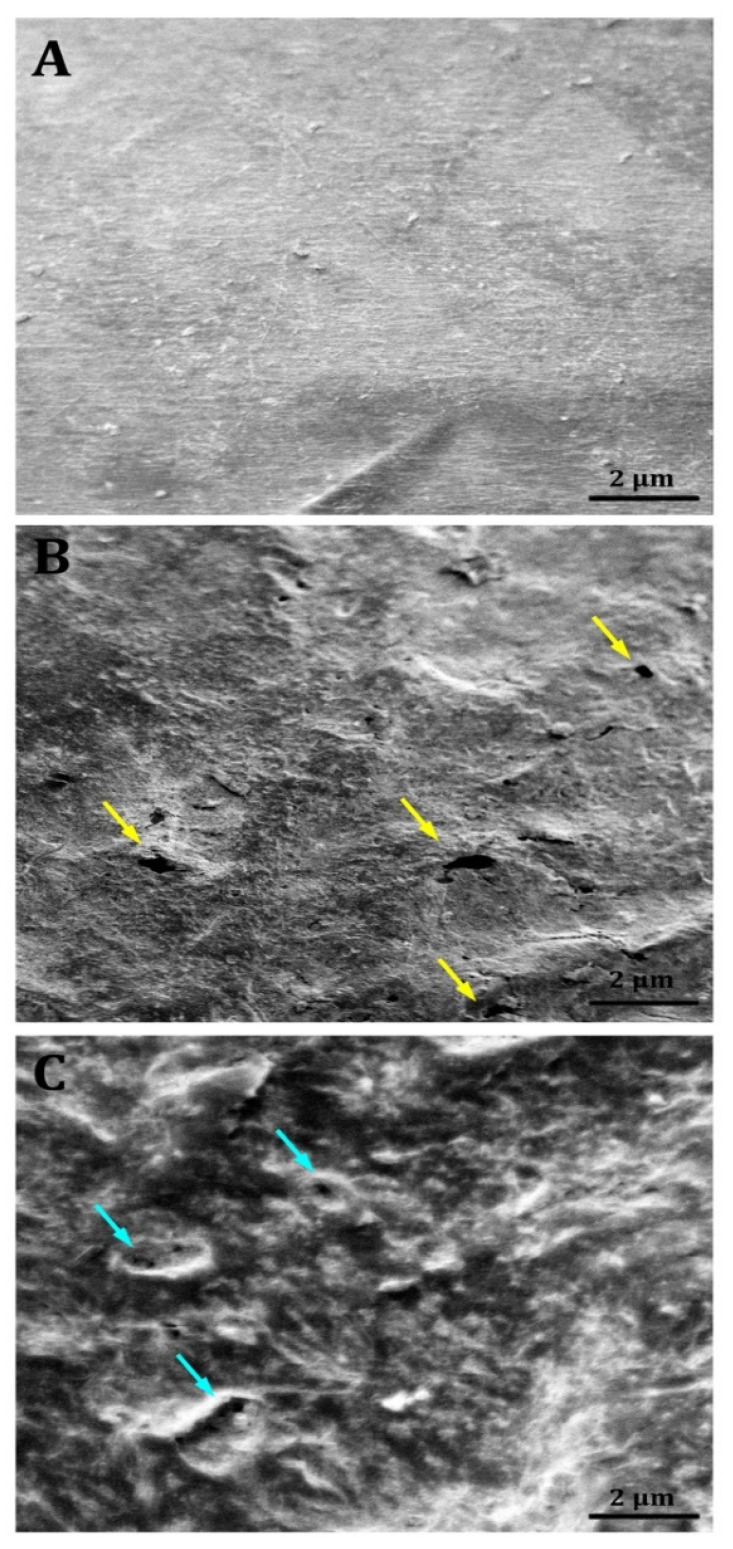
Scanning electron micrographs of LDPE sheets after 45 days of incubation: (**A**) Control LDPE sheet without bacterial treatment. (**B**) LDPE sheet treated with LDPE-DB2. (**C**) LDPE sheet treated with LDPE-DB26. Yellow arrows indicate the formation of cavities and pores resulting from LDPE-DB2 activity, while blue arrows highlight surface biofragmentation caused by LDPE-DB26.

Figure 6A illustrates that the control SEM micrographs exhibited no alterations in surface morphology, preserving a consistent surface throughout the experiment. This contrasted with the treated LDPE film samples. The SEM analysis demonstrated the biodegradation activity of LDPE-DB2 (Figure 6B) and LDPE-DB26 (Figure 6C) on the surface of LDPE. This process caused surface degradation marked by voids, cracks, and folds, among other characteristics. Sowmya et al. [63] reported that SEM analyses are the most effective method to confirm the degradation of plastic sheets by showing the formation of topographical changes after the biodegradation process. Zhang et al. [64] stated that cavity formation, a reduction in surface area, and a change in the surface physicochemical composition of the plastic indicate the effective biodegradation of plastic film. Hence, because of the degrading efficacy of LDPE-DB2 and LDPE-DB26 strains, these findings provide solid evidence for LDPE biodegradation with the advancement of LDPE-DB2 over LDPE-DB26.

#### 3.4.3. FTIR Analysis

**Figure 7:**
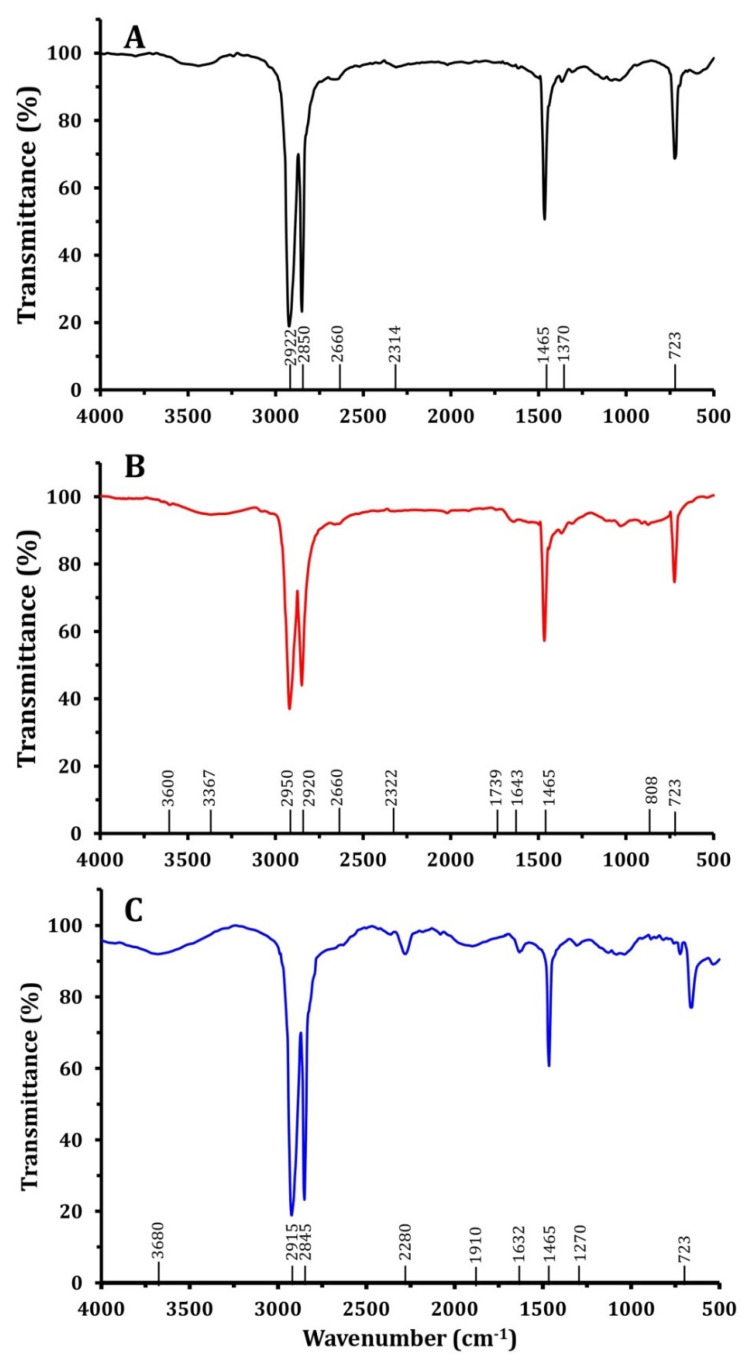
FTIR spectra show changes in the peaks of LDPE functional groups after 45 days of biodegradation compared with the untreated control: (**A**) FTIR spectrum of the control LDPE film. (**B**) LDPE film treated with LDPE-DB2. (**C**) LDPE film treated with LDPE-DB26.

FTIR spectroscopy was employed to investigate changes in the physicochemical composition of LDPE surfaces following biological pretreatment. Figure 7 presents the FTIR spectra of LDPE films before and after 45 days of biodegradation by *Bacillus cereus* LDPE-DB2 and *Pseudomonas aeruginosa* LDPE-DB26. Figure 7A represents the FTIR spectrum of untreated LDPE, showing characteristic peaks corresponding to polyethylene functional groups. The primary peaks include C–H stretching vibrations at 2922 cm^−1^ and 2850 cm^−1^, C–H bending at 1465 cm^−1^ and 1370 cm^−1^, and CH_2_ rocking at 723 cm^−1^. The absence of significant oxygen-containing functional groups (e.g., carbonyls or hydroxyls) confirms that the control LDPE is structurally intact and has not undergone oxidative degradation. Figure 7B shows the FTIR spectrum of LDPE films treated with *Bacillus cereus* LDPE-DB2. The emergence of new peaks suggests significant chemical modifications due to bacterial degradation. Notably, a strong peak appears at 1739 cm^−1^, indicating the formation of carbonyl (C=O) groups, which are associated with oxidation and polymer chain scission. Additionally, the peaks at 2322 cm^−1^ (C–C conjugation) and 808 cm^−1^ (C–H bending) further confirm polymer modification. The presence of hydroxyl-related peaks (3887 cm^−1^) suggests the introduction of hydrophilic functional groups, making the polymer surface more susceptible to microbial attack [65]. These results indicate that *Bacillus cereus* LDPE-DB2 facilitates extensive oxidative degradation, leading to structural breakdown. Figure 7C represents the FTIR spectrum of LDPE films treated with *Pseudomonas aeruginosa* LDPE-DB26. Similar to LDPE-DB2, new peaks appear, but with some differences in intensity and distribution. The 1270 cm^−1^ peak (C–O stretching) suggests the formation of esters or carboxylic acids, while the 1632 cm^−1^ peak (C=C stretching) indicates the presence of conjugated double bonds, likely due to oxidative cleavage of polyethylene chains. Additional peaks at 1910 cm^−1^ (C=O stretching) and 3680 cm^−1^ (O–H stretching) confirm polymer oxidation [66,67]. Although the degradation pattern is similar to that of LDPE-DB2, the intensity of carbonyl and hydroxyl peaks is lower, suggesting that *Pseudomonas aeruginosa* LDPE-DB26 exhibits a lower degradation efficiency compared with *Bacillus cereus* LDPE-DB2. The emergence of new functional groups (carbonyls, hydroxyls, and alkenes) in Figure 7B,C indicates microbial oxidation and polymer breakdown, with *Bacillus cereus* LDPE-DB2 showing stronger modifications than *Pseudomonas aeruginosa* LDPE-DB26, further supporting its higher LDPE degradation efficiency. These findings align with previous studies. Wasserbauer et al. [68] reported that *Pseudomonas putida* and *Bacillus brevis* oxidized polyethylene surfaces, producing carbonyl groups. In a study by Arutchelvi et al. [69], the formation of carbonyl (-C–O) groups was observed during PE biodegradation, which is considered as a crucial step in the process. Additionally, Chakraborty et al. [70] observed carbonyl group formation during polyethylene biodegradation, a critical step in the degradation process. Yang et al. [71] utilized micro-ATR/FTIR and XPS analysis and found the formation of carbonyl group peaks in plastic samples treated with *Bacillus* sp. and *Enterobacter asburiae*, demonstrating that these strains can oxidize PE structures, leading to carbonyl group formation and subsequent surface deterioration and fragmentation. Similarly, other studies have reported detectable structural changes in LDPE films after microbial treatment, as observed by FT-IR spectroscopy [72]. During the biodegradation process, microbial enzymes—particularly oxidative enzymes such as laccases, peroxidases, and monooxygenases—can catalyze oxidation and other transformations that lead to the formation of functional groups (e.g., hydroxyl, carbonyl, and carboxyl) on the LDPE surface. These chemical modifications contribute to polymer chain scission, surface deterioration, and increased susceptibility to further microbial attack.

#### 3.4.4. XRD Analysis

**Figure 8:**
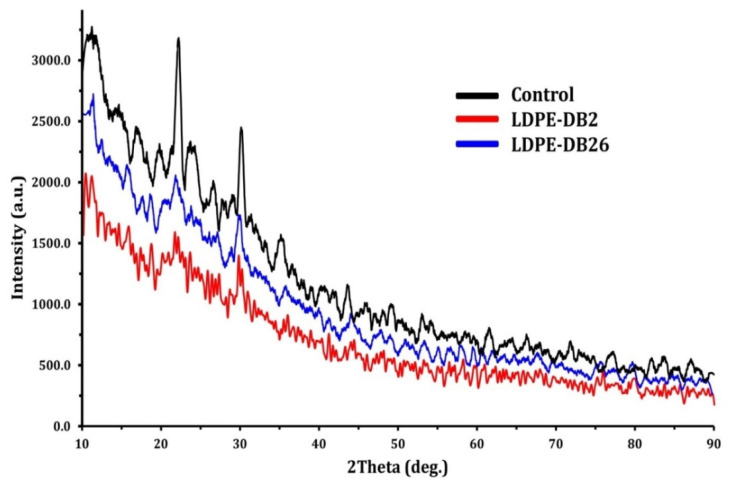
XRD analysis of LDPE film before and after treatment using the selected bacterial strains LDPE-DB2 and LDPE-DB26.

Figure 8 presents the XRD spectra of LDPE films before and after 45 days of biodegradation by *Bacillus cereus* LDPE-DB2 and *Pseudomonas aeruginosa* LDPE-DB26, with the untreated control. The XRD spectra of the control LDPE exhibit characteristic sharp peaks, indicating a higher degree of crystallinity in the untreated plastic. However, after microbial treatment, a noticeable reduction in peak intensity is observed for both bacterial strains, suggesting a decrease in crystallinity due to polymer degradation. The reduction is more pronounced in LDPE-DB2-treated films, as seen from the lower intensity of diffraction peaks compared with LDPE-DB26-treated films, indicating that *Bacillus cereus* LDPE-DB2 caused a greater disruption of the crystalline structure of LDPE. The untreated LDPE film exhibited distinct peaks at 11.24°, 16.8°, 22.22°, 23.27°, 26.9°, 30.14°, 35.16°, 43.58°, 49.12°, and 53.02°. In contrast, the LDPE film treated with LDPE-DB2 showed distinct peaks at 10.28°, 11.96°, 15.68°, 18.64°, 21.6°, and 29.72°, while the film treated with LDPE-DB26 displayed peaks at 11.24°, 15.52°, 18.46°, 21.72°, 29.74°, 35.74°, 43.98°, and 47.88°. The variation in peak intensity, width, arrangement, and formation indicates that both LDPE-DB2 and LDPE-DB26 effectively degraded the LDPE film. These changes suggest that biological treatment altered the crystallinity of the LDPE [12]. XRD spectrum analysis revealed that the initial crystallinity of untreated LDPE was 52.3%. After 45 days of biodegradation, crystallinity decreased to 42.7% with LDPE-DB2 treatment (a reduction of 18.3%) and to 34.7% with LDPE-DB26 treatment (a reduction of 33.7%). Similar results were reported by Gupta and Devi [18], who observed a 23% reduction in LDPE crystallinity after 60 days of incubation with *Pseudomonas aeruginosa* ISJ14. In conclusion, the reduction in peak intensity is more pronounced in LDPE-DB2-treated films, suggesting that *Bacillus cereus* LDPE-DB2 induced greater polymer disruption and structural modifications compared with LDPE-DB26. The decrease in crystallinity is associated with enzymatic oxidation and hydrolysis of polymer chains, leading to an increase in amorphous regions within the polymer matrix. This change facilitates further microbial degradation, as amorphous regions are more accessible to enzymatic attack than crystalline regions.

### 3.5. Proposed Mechanism of LDPE Degradation by Bacteria

The metabolic profile of biodegraded LDPE is shown in Table 1. After LDPE-DB2 biodegradation, the GC-MS data showed 12 byproducts including long-chain alkanes (e.g., tridecane and octacosane), small-chain alkanes (e.g., butane), alkenes (e.g., 1-heptene, 1,3,5,7-cyclooctatetraene, 1-decene, and 1-nonadecene), carboxylic acid (e.g., acetic acid), aldehyde (e.g., lauric aldehyde), alcohols (e.g., n-dodecan-1-ol and 1-octadecanol), and amine (e.g., N,N-dimethyltetradecylamine). However, the GC-MS data of LDPE treated with LDPE-DB26 showed nine byproducts, including long-chain alkanes (e.g., undecane), alkenes (e.g., 1-decene, heptadecane, and 1-nonadecene), carboxylic acids (e.g., lauric acid, propanoic acid, tetradecanoic acid, and octadecanoic acid), and alcohol (e.g., 1-octadecanol). The proposed mechanism for LDPE biodegradation by the isolated bacterial strains is based on the integration of FTIR, XRD, and GC-MS data, supported by their enzymatic activities and literature evidence. Initially, oxidative enzymes such as Lac and LiP act on the high-molecular-weight, hydrophobic LDPE surface [73,74]. These enzymes introduce oxygen-containing functional groups (e.g., hydroxyl, carbonyl), as evidenced by FTIR peaks at 1739 cm^−1^ and 1030 cm^−1^, corresponding to carbonyl (C=O) and hydroxyl (C–O) stretching vibrations. This oxidation process disrupts the polymer’s hydrophobicity, increases surface polarity, and enables subsequent microbial colonization and biofilm formation [18,75]. XRD analysis revealed a measurable decrease in crystallinity after 45 days of microbial treatment, confirming structural disruption of the polymer matrix [76]. SEM images further support this by showing visible surface erosion, cavities, and micro-fractures, consistent with polymer degradation [77]. Following initial oxidation, the polymer chains undergo cleavage, producing short-chain hydrocarbons (e.g., tridecane, undecane, heptadecane) identified by GC-MS. These compounds undergo further oxidative transformation into more polar intermediates such as alcohols (1-octadecanol, n-dodecanol), aldehydes (e.g., lauric aldehyde), and carboxylic acids (e.g., acetic, propanoic, lauric, and myristic acids). The formation of these products is consistent with a stepwise degradation model involving oxidation, dehydrogenation, and β-oxidation-like processes [78,79]. Some ester compounds (e.g., ethyl propionate) were also detected, possibly formed via microbial esterification of acids and alcohols under metabolic or storage conditions. Notably, the detection of N,N-dimethyltetradecylamine, an amine, likely reflects microbial metabolic byproducts rather than direct transformation from carboxylic acids, correcting previous mechanistic oversimplifications. Ultimately, the bacterial metabolism likely channels these intermediate products into central metabolic pathways, leading to mineralization into CO_2_ and H_2_O, as reflected by the reduction in molecular weight and the decrease in film mass. This mechanistic sequence supports a model in which oxidative enzymatic action initiates polymer destabilization, followed by microbial enzymatic metabolism of fragmented products. The process is not driven by a single dominant pathway but rather a sequential and complementary cascade supported by bacterial enzymatic systems capable of processing diverse hydrocarbons and functionalized intermediates.

The toxicity of the degradation byproducts was assessed and summarized in Table 2 [80,81,82,83,84,85,86,87,88,89,90,91,92,93,94]. Non-halogenated alkanes, such as undecane, tridecane, and heptadecane, exhibit extremely low toxicity [17]. Alkanes and other hydrophobic compounds typically have limited water solubility, reducing their bioavailability and potentially harmful effects [95]. For example, the acute oral LD_50_ for dodecane is 2000 mg/kg, and for complex alkane mixtures (C_10_–C_17_), LD_50_ values range from 2000 to 5000 mg/kg [96]. Alkanes like decane and undecane are widely used in engine and jet fuels, solvents, and scientific research, with extensive safety evaluations [97]. Carboxylic acids, such as lauric acid, are commonly used in the food industry as additives, flavoring agents, and nutritional supplements [98]. The use of bacterial strains for LDPE biodegradation is a promising approach due to its cost-effectiveness, environmental friendliness, reduced contaminant production, and generation of non-toxic metabolites.

The feasibility of applying *Bacillus cereus* LDPE-DB2 and *Pseudomonas aeruginosa* LDPE-DB26 for industrial-scale LDPE biodegradation is promising but requires further optimization. Although *Bacillus cereus* LDPE-DB2 demonstrated a higher degradation rate (19.8% weight loss in 45 days) compared with *Pseudomonas aeruginosa* LDPE-DB26 (11.6%), large-scale applications demand enhanced bacterial growth, biofilm formation, enzymatic activity, and biodegradation kinetics. Strategies such as genetic modification, co-culturing with other plastic-degrading microorganisms, or enzyme engineering could further improve efficiency [30]. One of the key advantages of *Bacillus cereus* LDPE-DB2 is its strong biofilm formation, which enhances bacterial adhesion to LDPE surfaces and prolongs enzymatic activity. This characteristic could be leveraged in biofilm reactors to maximize bacterial colonization and improve degradation efficiency in industrial settings. The study also highlights the role of extracellular oxidative enzymes such as Lac, LiP, and MnP, which could be utilized in enzyme-based degradation approaches rather than relying on live bacterial cultures. This would allow for faster and more controlled polymer breakdown while mitigating concerns regarding bacterial viability and contamination. Industrial applications would require scalable bioreactor systems optimized for pH, temperature, and oxygen conditions to ensure maximum bacterial activity and polymer degradation efficiency. Additionally, integrating microbial degradation with mechanical or chemical pretreatments (e.g., oxidation or thermal degradation) could significantly accelerate LDPE breakdown. However, environmental and economic considerations must be addressed before large-scale implementation. The cost-effectiveness of cultivating bacterial strains, producing enzymes, and maintaining sustainable processes is critical for industrial feasibility. Additionally, potential risks associated with the release of genetically modified or engineered bacteria into natural ecosystems must be carefully assessed [99]. Controlled biodegradation facilities or closed-system bioreactors would be necessary to minimize unintended ecological consequences. Future research should focus on optimizing bacterial strains, improving enzyme efficiency, and designing scalable bioreactor systems to facilitate the industrial application of LDPE biodegradation. While *Bacillus cereus* LDPE-DB2 and *Pseudomonas aeruginosa* LDPE-DB26 show strong potential for microbial-based plastic degradation, further advancements in bioprocess optimization, enzyme engineering, and risk assessment are essential for their successful large-scale application. By leveraging microbial enzymes and insect gut symbionts, novel biotechnological solutions can be developed to mitigate global plastic waste accumulation.

## 4. Conclusions

This study showed that *B. cereus* LDPE-DB2 and *P. aeruginosa* LDPE-DB26, which were found in the guts of waxworms and termites, can break down LDPE film effectively. Both types of bacteria were able to stick to the LDPE surface and form biofilms. However, LDPE-DB2 had much better biofilm growth and was less likely to stick to water than LDPE-DB26. When incubated for 45 days, LDPE films treated with LDPE-DB2 and LDPE-DB26 exhibited a noticeable weight reduction of 19.8% and 11.6%, respectively, compared with the control films. Kinetic analysis showed that LDPE-DB2 shortened the half-life of biodegradation to 73.48 days, while LDPE-DB26 shortened it to 178.5 days. In terms of mechanical properties, LDPE-DB2 treatment led to a higher reduction of 58.3% in TS compared with 43.1% for LDPE-DB26 treatment. Characterization techniques provided evidence that both strains were able to partially degrade the LDPE polymer structure. LDPE-DB2 treatment showed greater decreases in molecular weight and crystallinity compared with LDPE-DB26 treatment. After biological treatment, SEM and FTIR tests showed that the surface of the LDPE films had erosion and holes, and functional groups had formed. Our findings not only provide new insights into the potential of insect-associated bacteria to degrade LDPE but also contribute to closing key knowledge gaps in the field by addressing both the environmental distribution of LDPE-degrading microbes and the stepwise enzymatic pathways involved in polymer breakdown. While the results demonstrate promising LDPE biodegradation by insect-gut-derived bacterial strains, this study has certain limitations. The biodegradation experiments were conducted under controlled laboratory conditions, which may not fully represent environmental complexities. Additionally, while key metabolic intermediates were identified, the complete enzymatic pathways and regulatory mechanisms remain to be fully elucidated. Further research involving in situ studies, transcriptomic/proteomic analyses, and long-term degradation assessments will be necessary to validate and extend these findings.

## Figures and Tables

**Table 1:** GC-MS identification of intermediate products of LDPE biodegradation by bacterial strains.

No.	RT (min)	Compound Name	Molecular Formula	*m*/*z*	Mw	Classification
LDPE-DB2						
1	1.62	Butane	C_4_H_10_	43	58	Alkane
2	3.64	1-Heptene	C_7_H_14_	41	98	Alkene
3	4.34	Acetic acid	C_2_H_4_O_2_	43	60	Carboxylic acid
4	6.94	1,3,5,7-Cyclooctatetraene	C_8_H_8_	104	104	Alkene (cyclic)
5	8.62	1-Decene	C_10_H_20_	41	140	Alkene
6	13.51	Tridecane	C_13_H_28_	57	184	Alkane
7	13.16	Lauric aldehyde	C_12_H_24_O	43	184	Aldehyde
8	15.42	n-Dodecan-1-ol	C_12_H_26_O	55	186	Alcohol
9	17.66	N,N-Dimethyltetradecylamine	C_16_H_35_N	58	241	Amine
10	20.51	1-Nonadecene	C_19_H_38_	55	266	Alkene
11	21.13	1-Octadecanol	C_18_H_38_O	83	270	Alcohol
12	26.76	Octacosane	C_28_H_58_	57	394	Alkane

Mw, molecular weight; *m*/*z*, mass/charge (*m*/*z*); RT, retention time (min).

**Table 2:** Toxicity of the detected metabolites after biodegradation assay.

Compound	LD_50_ (mg/kg)	References
1-Decene	Low toxicity	[80]
1-Heptene	Low toxicity	[81]
1-Nonadecene	Low toxicity	[81]
1-Octadecanol	>2000	[82]
Acetic acid	3530	[83]
Heptadecane	>5000	[84]
Lauric acid	>10,000	[85]
N,N-Dimethyltetradecylamine	1320	[86]
n-Dodecan-1-ol	Non-toxic	[87]
Lauraldehyde	23,000	[88]
Octacosane	1000	[89]
Propanoic acid	Low toxicity	[90]
Myristic acid	>5000	[91]
Tridecane	>5000	[87]
Undecane	>2000	[87]
Ethyl propionate	>2000	[92]
1-Octadecanol	>2000	[93]
1,3,5,7-Cyclooctatetraene	Low toxicity	[94]

LD, lethal dose.

## Data Availability

All datasets generated for this study are included in the article.
